# Eating Behavior and Eating Habits: From Infancy to Adolescence

**DOI:** 10.3390/nu18122000

**Published:** 2026-06-19

**Authors:** Ivie Maneschy, María L. Miguel-Berges, Andrea Jimeno-Martínez, Guiomar Masip, Luis A. Moreno

**Affiliations:** 1 Growth, Exercise, NUtrition and Development (GENUD-B34_23R) Research Group, Instituto Agroalimentario de Aragón (IA2), Universidad de Zaragoza, Instituto de Investigación Sanitaria de Aragón (IIS Aragón), 50009 Zaragoza, Spain; ireis@unizar.es (I.M.); mlmiguel@unizar.es (M.L.M.-B.); a.jimeno@unizar.es (A.J.-M.); gmasip@unizar.es (G.M.); 2 Centro de Investigación Biomédica en Red de Fisiopatología de la Obesidad y Nutrición (CIBERObn), Instituto de Salud Carlos III, 28029 Madrid, Spain

**Keywords:** eating behaviors, early-life nutrition, dietary patterns, appetitive traits

## Abstract

Eating behavior and eating habits are shaped from the earliest stages of life through interactions among biological, familial, social, and environmental factors. The aim of this narrative review is to integrate evidence on the early-life determinants of eating behavior and their influence on dietary intake from infancy to adolescence. A narrative review was conducted with a structured search approach prioritized on longitudinal studies, intervention trials, and policy evaluations when available, and using cross-sectional evidence mainly to describe patterns and sociodemographic factors. Synthesizing the current evidence, our framework proposes that breastfeeding, responsive complementary feeding, and self-regulatory parenting are associated with higher responsiveness to internal hunger, satiety cues, and preference for nutrient-dense foods. Conversely, coercive practices, early exposure to highly palatable foods, and the influence of food marketing are linked to dominant hedonic responses and impulsive consumption patterns. Furthermore, family environments characterized by stress or food insecurity, together with high access to low-nutrient foods, may increase vulnerability to poor eating habits and emotional eating during adolescence. Overall, the evidence highlights the need for preventive interventions that integrate parenting support, school food education, digital marketing regulation policies, and the promotion of healthy food environments across multiple sectors. Understanding the biological, psychological, and social factors linking early determinants to dietary intake and eating behaviors across development is essential for promoting a balanced relationship with food and preventing chronic diseases from an early age.

## 1. Introduction

Eating behavior patterns and eating habits during childhood and adolescence account for a substantial proportion of the variation in cardiometabolic health, particularly through obesity development [1,2,3,4,5]. To ensure conceptual rigor, this review distinguishes between eating behaviors, defined as individual psychological and physiological responses to food stimuli, and eating habits, which constitute acquired and repetitive sociocultural routines of food consumption [6,7]. These constructs are fundamentally underpinned by appetitive traits, biologically based predispositions toward food approach or avoidance. They are further modulated by appetite self-regulation as the capacity to adjust intake according to internal satiety cues [8,9]. Furthermore, we differentiate between dietary intake, referring to actual energy and nutrient consumption, and diet quality, which reflects the alignment of such intake with health-promoting nutritional standards [4,5,7,10,11]. Beyond these cardiometabolic implications, such multifaceted behaviors and habits are also associated with mental health outcomes, such as depression [12,13,14]. From early childhood, the family environment may influence dietary patterns and children’s appetite traits through parenting practices, home food availability, eating habits and emotional dynamics during mealtimes [15,16,17]. These factors, together with genetic predisposition [18,19] and societal influences related to the food environment, contribute to the development of preferences and habits that tend to persist throughout growth.

These behaviors also emerge in environments characterized by the extensive availability, relatively low cost, and intensive marketing of energy-dense, low-nutrient foods, which influence dietary norms from an early age [20,21,22]. Early experiences, such as the duration and exclusivity of breastfeeding, responsive complementary feeding, and parenting styles, are linked to the development of appetite self-regulation and food preferences [15,23,24,25,26]. These patterns tend to persist into adolescence, linking early family and environmental factors to eating habits and the risk of obesity and other cardiometabolic disorders [10,18,27].

Children’s eating behaviors can be grouped into two categories: food approach and food avoidance behaviors. The former reflects a greater tendency to seek out or respond positively to food stimuli (e.g., enjoyment of food or food responsiveness), whereas the latter is associated with a tendency to limit intake or reject certain foods (e.g., satiety responsiveness or food fussiness). The most recent evidence shows that food approach behaviors are associated with higher intake of fruit and vegetables, as well as higher intake of foods rich in fat, sugar, or salt, particularly in obesogenic environments. In contrast, food avoidant subscales are associated with lower overall food and energy intake, with possible variation by age and gender [28,29].

Although previous reviews have focused on specific developmental stages or specific determinants, few have addressed how early determinants from infancy to adolescence jointly shape eating behaviors and eating habits across development. Despite advances in our understanding of biological and family factors, the available evidence remains fragmented and rarely considers biological, psychological and environmental factors within a common developmental framework.

The core focus of this review is the development of eating behaviors and eating habits across childhood and adolescence, with particular attention to early-life determinants from the prenatal period to adolescence. Genetic, familial, school-based, community, digital, and policy-related factors are discussed as part of the broader ecological context surrounding this developmental process.

The breadth and complexity of eating behavior development from infancy to adolescence, together with the interplay of biological, familial, social, environmental, and policy-level determinants, make this topic particularly suitable for an interpretive and integrative synthesis. The available literature is heterogeneous in design, populations, measurement instruments, developmental stage, and level of analysis, with evidence drawn from observational, interventional, and policy studies that address different but complementary dimensions of the phenomenon. For this reason, a narrative review can be especially helpful because it allows integration, interpretation, and critical appraisal of evidence that may not be easily summarized within a single framework.

Hence, this review aims to address this gap by critically analyzing early-life determinants of eating behaviors and eating habits from infancy to adolescence. Adopting an integrative, developmental perspective, this review seeks to consider biological, familial and socioeconomic factors to inform clinical practice, educational programs and public policy. Given the heterogeneity of study designs in this field, most of the available evidence does not allow causal conclusions. Therefore, this manuscript distinguishes between associations and effects, interpreting pathway narratives as conceptual frameworks unless supported by randomized or strong quasi-experimental evidence.

## 2. Review Methodology

In accordance with established narrative review methodologies [30], this review adopts an interpretivist approach rather than a neutral compilation of literature. The underlying assumption is that eating behaviors are shaped by a continuous, dynamic interaction between innate biological predispositions and evolving modern structural environments. This perspective directly influenced the emphasis placed on digital and policy determinants in this review.

To ensure a comprehensive literature search and build this author-driven conceptual model, an iterative strategy was employed across PubMed, Web of Science, Embase and Scopus. The search primarily focused on peer-reviewed studies published between 2010 and 2025. However, seminal papers and foundational literature published prior to this timeframe were selectively included due to their historical relevance and ongoing impact on the field.

The initial broad search employed key terms related to the life-course approach (“responsive feeding”, “appetitive traits”, “parenting styles”, and “food environment”), in combination with “eating habits” and “eating behavior”.

During the review process, the scope was iteratively refined. Regarding the literature selection process, we employed a purposive sampling designed to support our developmental framework. Rather than aiming for exhaustive quantitative extraction, our goal was to achieve conceptual sufficiency to adequately substantiate each axis of the model. To this end, the selection of evidence was guided by a hierarchical approach. We purposefully included peer-reviewed original research, prioritizing longitudinal cohort studies and randomized controlled trials (RCTs) to better establish temporal associations, as well as recent systematic reviews and large-scale policy evaluations. Conversely, we explicitly omitted non-peer-reviewed preprints, opinion pieces, and animal-based studies, as our objective was to synthesize evidence concerning human developmental trajectories. Furthermore, the material was narrowed by excluding studies focused primarily on clinical eating disorders (e.g., anorexia nervosa) to maintain a clear focus on normative eating behaviors and obesity risk. At the same time, the search was expanded to include literature on digital food environments and structural policy interventions, as early findings indicated their growing influence on adolescent eating behaviors. Finally, the retrieved literature was identified and reviewed by the lead author, with the final selection and integration of evidence validated through consensus among all co-authors. This process aimed to minimize selection bias and ensure that the most representative and high-quality evidence was included for each developmental stage.

### Data Synthesis and Integration

To translate the retrieved literature into our final synthesis, a thematic approach was employed. The extracted evidence was systematically grouped and analyzed through a dual lens: a socio-ecological framework (ranging from individual neurobiology to macro-level policies) and a life-course perspective (from the prenatal period through adolescence).

**Figure 1:**
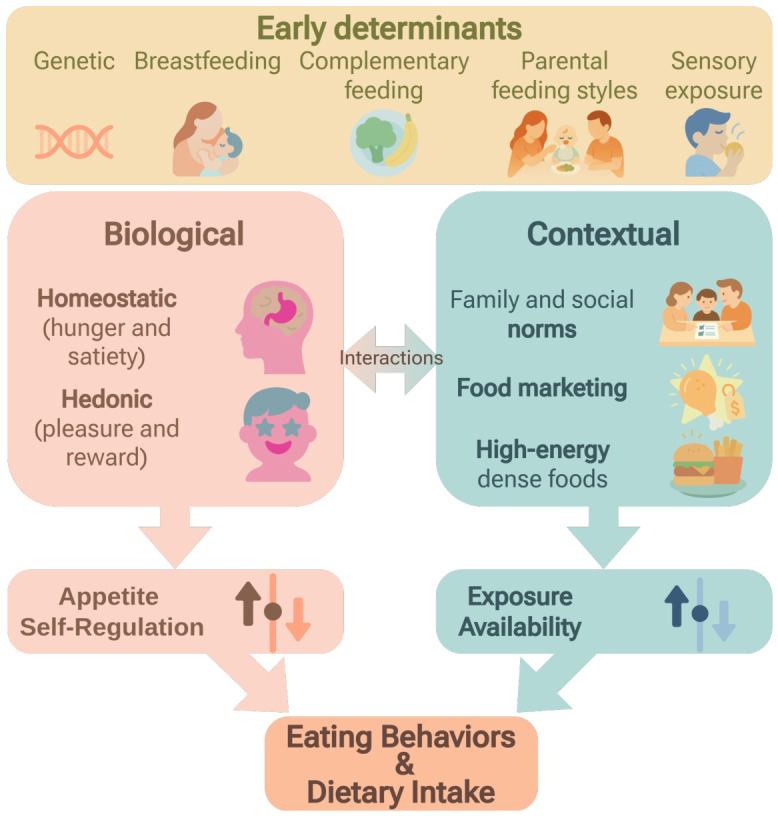
Neurobiological and contextual integration of eating behavior and dietary intake. Vertical arrows represent hypothesized developmental pathways, while the central horizontal arrow highlights the bidirectional interaction between internal biological (homeostatic–hedonic) systems and contextual environmental cues. Together, these dynamic links, primarily derived from observational and neuroimaging data, shape appetite self-regulation and ultimate dietary outcomes.

**Figure 2:**
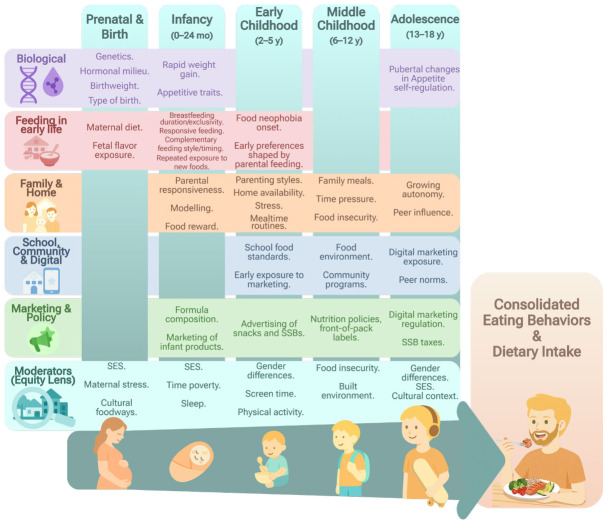
Developmental trajectory of determinants of eating behavior and dietary intake. Factors represent key associations identified in the literature across the life course, from the prenatal period to adolescence. The horizontal arrow illustrates the cumulative and progressive nature of these influences, which converge into the consolidated eating patterns observed in late adolescence. Strength of evidence varies by determinant and study design. SES = socioeconomic status SSB = Sugar-sweetened beverages.

The development of our main interpretive axes and figures followed a structured logic. First, to construct the biological–environmental axis (Figure 1), literature concerning internal appetite regulation was mapped against external food exposure findings. This allowed us to interpret eating behavior as the intersection of homeostatic-hedonic drivers and contextual cues. Second, to trace developmental trajectories (Figure 2), the grouped evidence was organized into a temporal matrix, assigning specific determinants (e.g., responsive feeding, school policies) to their corresponding critical periods of development. Finally, the logic behind assigning meaning to these pathways relied on identifying recurrent themes in the literature—specifically, how early psychosocial and biological predispositions are repeatedly shown to interact with evolving structural environments. This structured interpretive process ensured that the final narrative was directly grounded in the logical grouping of the reviewed evidence.

## 3. Conceptual Framework

In this review, eating behaviors are primarily described through validated psychometric instruments, with a focus on food approach and food avoidance dimensions as assessed by the Children’s Eating Behaviour Questionnaire (CEBQ) [9] and the Dutch Eating Behaviour Questionnaire (DEBQ) [31]. Eating behaviors are shaped from the earliest stages of development by a complex interplay of biological, psychological, and social factors, which influence the processes for appetite self-regulation and sensitivity to food stimuli [10,32]. These factors include early feeding experiences such as breastfeeding, responsive complementary feeding, parenting styles, and sensory exposure. Together, they contribute to the development of appetite-related traits [15,33].

At a neurobiological level, eating behaviors are regulated by the integration of the homeostatic and hedonic systems, which coordinate physiological and emotional responses to food [34,35,36]. This integration is influenced by brain maturation, particularly in the hypothalamus, amygdala and prefrontal cortex, within family and social contexts that shape food availability [32,37].

### 3.1. Regulation of Eating Beaviors

Over recent years, an increasing body of evidence has shown that eating behaviors involve complex brain-regulation processes that integrate homeostatic, hedonic and cognitive signals. These processes determine how internal and external food-related cues are perceived, interpreted and responded to [38,39].

Building on this body of evidence, Figure 1 provides a contextual synthesis in which early determinants of eating behaviors are organized around two interrelated axes. The first axis reflects biological systems involved in appetite regulation, and the second captures contextual environmental factors shaping food exposure and availability from the earliest stages of development. To move beyond a linear interpretation, this model explicitly highlights the bidirectional interactions between these biological and contextual systems. This dynamic interplay illustrates how early determinants shape the development of appetite self-regulation, ultimately resulting in specific eating behaviors and dietary intake.

#### 3.1.1. Homeostatic–Hedonic Regulation Axis

This axis captures the combined contributions of the homeostatic system, which maintains energy balance through hunger and satiety signals, and the hedonic system, which is oriented towards the pleasure and reward associated with food [35,39,40].

The development of self-regulation is more likely to occur in the context of responsive parenting practices, stable eating routines, and early and positive exposure to healthy foods [41,42,43]. However, the predominance of the hedonic system, reinforced by the high availability of energy-dense foods, dopaminergic reward processes and marketing cues, may be associated with dysregulated eating patterns and reduced sensitivity to internal satiety signals, although the magnitude and direction of these associations vary across contexts and study designs [34,36,40].

During the high-brain-plasticity stages of childhood and adolescence, striking a balance between the two systems is essential for developing healthy eating behaviors [32,37].

#### 3.1.2. Environmental Influence and Food Learning Axis

This axis describes how physical, familial, and social environments are linked to the expression of appetitive traits and food choices [7,44].

- Repeated exposure to different foods and positive sensory experiences promotes dietary acceptance and diversification, favoring the development of stable preferences for healthy options [42].
- Food availability is determined by the food supply, economic factors, physical access to food, household norms and cultural or media influences surrounding food [45,46].

Both components act synergistically. High exposure to healthy foods within an accessible and structured environment facilitates self-regulation. Conversely, the abundant availability of high-energy-dense, low-nutrient dense foods in disorganized or stressful contexts reinforces the search for immediate pleasure and reduces the ability for appetite self-regulation [44,45]. Furthermore, eating behaviors can be understood as a learned behavior acquired through observation and experience. Eating is a socially shaped skill influenced by early experiences, family practices and the wider cultural environment [7,47].

## 4. Evidence Across Developmental Stages and Determinants

Viewed through our interpretive lens, we conceptualize the development of eating behaviors and dietary intake as a continuous process that begins before birth and consolidates during childhood and adolescence. Growing evidence indicates that this process is shaped by biological, familial, social, and cultural factors. Early determinants play a central role in shaping appetite trajectories, eating preferences and eating patterns [7,48,49].

### 4.1. Genetic Determinants

Twin studies have shown that eating behaviors are characterized by both genetic and environmental factors. Studies of large twin cohorts, such as the UK’s Gemini Study and Twins Early Development Study (TEDS) [50], as well as the Finnish FinnTwin12 and FinnTwin16 cohorts [51,52], have reported moderate to high heritability estimates for various eating behaviors, such as food responsiveness, satiety responsiveness and enjoyment of food, ranging from 40% to 75% [19,49,53,54,55]. Specifically, research in young adults has shown significant heritability for uncontrolled eating, cognitive restraint, and emotional eating [52], as well as for distinct patterns like snacking, infrequent and unhealthy eating, and avoidant eating [51].

In addition to these traits, twin studies have shown that the associations between eating behaviors and obesity indicators (such as BMI or waist circumference) are also explained by both genetic and environmental factors [51,52]. Moreover, these traits may predict energy intake when children are exposed to environments rich in energy-dense, highly palatable foods [49,56,57,58]. This gene–environment interplay suggests that heritable appetitive traits may be linked to a higher vulnerability to obesogenic environments, whereas supportive environments, such as structured mealtime routines and responsive feeding, represent key modifiable environmental factors [58,59].

Genome-wide association studies (GWAS) have identified millions of genetic variants associated with BMI (Body Mass Index). Most of the genetic variants are expressed in the central nervous system, such as the *FTO* (fat mass and obesity-associated) gene, which has been associated with increased neural activation in the striatum and orbitofrontal cortex when exposed to palatable foods, indicating a heightened dopaminergic reward response [60,61,62]. Similarly, the *MC4R* (melanocortin 4 receptor) gene has been implicated in dysregulated satiety signaling and higher susceptibility to overeating [63]. Findings from mediation analyses have shown that eating behaviors partly explain the genetic susceptibility to obesity [64]. Furthermore, BMI-associated genetic variants have also been associated with eating behaviors, with this association being explained to a greater extent by obesity indices [65,66], suggesting genetic overlap between BMI and eating behaviors. Recent neuroimaging–genetic studies have shown that genetic variation interacts with environmental factors, such as parental feeding style, to influence brain regions involved in motivation and inhibitory control, thereby shaping the long-term development of eating behaviors [19].

### 4.2. Pre-And Peri-Natal Determinants

During the prenatal period, maternal nutrition, gestational metabolism and exposure to hormonal and environmental factors are associated with the development of systems related to appetite and energy regulation [67]. An imbalanced nutrient intake or metabolic alterations during pregnancy can affect leptin and insulin sensitivity, which have been linked to an increased risk of being overweight and a heightened hedonic response to food later in life [68].

Birth weight and accelerated early postnatal growth also predict differences in eating behaviors: infants with rapid weight gain tend to show greater responsiveness to food and lower sensitivity to satiety from early infancy [58,69]. This evidence suggests that early metabolic patterns may predispose individuals to less efficient appetite regulation early in life.

### 4.3. Breastfeeding and Complementary Feeding

Breastfeeding has been associated with the development of appetite self-regulation, potentially by supporting responsiveness to hunger and satiety cues [48]. Several longitudinal studies have observed that children who are breastfed exclusively and for a prolonged period have greater responsiveness to satiety, engage in less emotional eating and are at a lower risk of developing obesity [18,24,70,71].

During the transition to complementary feeding, practices based on responsiveness, such as observing the child’s cues, providing a variety of options, and repeated exposure to natural flavors, have been associated with greater acceptance of fruits and vegetables, as well as with improved appetite self-regulation [42,43]. Evidence also suggests that responsive complementary feeding practices are linked to a lower risk of early obesity, highlighting the importance of parental guidance during this period [72]. In contrast, coercive practices, such as using food as a reward or the early introduction to highly palatable foods, are associated with greater impulsive eating patterns and a more emotional bond with food [20,73].

### 4.4. Family Environment and Parenting Styles

During childhood, family and caregiving contexts remain central spaces where food routines, modelling and mealtime dynamics are learned. At the same time, these contexts are highly heterogeneous, including single-parent households, shared custody, extended family care and families facing chronic stress, time poverty, or food insecurity. These conditions can constrain feasibility and modify how feeding practices translate into eating habits [6,7,74].

Authoritative parenting styles, characterized by emotional support and clear boundaries, are associated with better self-regulation [15,75]. In contrast, restrictive or indulgent parenting styles are linked to greater hedonic responsiveness and lower satiety sensitivity [41,76]. Children who are exposed to parental models who enjoy healthy foods are more likely to develop a preference for fruits and vegetables, highlighting the importance of vicarious learning in forming eating habits [42,48,77].

Overall, the emotional climate at home and the consistency of parental modelling are associated with children’s capacity to respond appropriately to hunger and satiety cues [6,15]. However, in our synthesis, we argue that these relationships are inherently bidirectional and contextual. Rather than a simple top-down influence from caregivers to children, our model emphasizes a reciprocal dynamic where children’s innate appetitive traits and temperament actively shape parental feeding practices [78,79]. For instance, children exhibiting high food responsiveness may elicit more restrictive control from parents, while those with pronounced food fussiness may prompt caregivers to employ greater pressure-to-eat strategies to ensure adequate intake [76,80]. This complex interaction highlights that parental practices often emerge as a response to the child’s specific behavioral profile, a perspective that is crucial for a nuanced understanding of how eating habits consolidate across development [33,81].

### 4.5. School, Community, and Digital Environments

As children grow, the context of food socialization expands to include school, the community, and the digital environment. School feeding programs that provide access to fruits, vegetables, and balanced meals are linked with sustained improvements in diet quality. In these contexts, outcomes are typically assessed through changes in daily servings of fruits and vegetables, often measured via 24-h dietary recalls, plate-waste assessments or Mediterranean diet adherence questionnaires, as well as through the availability of competitive foods (e.g., snacks and sugar-sweetened beverages) within school premises [11,82,83,84].

Community environments also have an important influence. For example, families living in neighborhoods with a high concentration of convenience stores, also known as ‘food swamps’, have greater exposure to energy-dense foods and lower dietary diversity [85,86]. Conversely, urban environments with local policies promoting access to fresh food and community kitchens are associated with healthier habits. These influences are frequently measured through observational studies assessing peer modeling, where children’s acceptance of novel or healthy foods is quantified based on the intake of peers in social settings, as well as the implementation of mandatory nutritional standards for school-distributed meals [82,87].

The digital environment has also emerged as an increasingly relevant factor. Exposure to advertising for highly palatable foods on social media and audiovisual platforms increases preference for sugary and salty foods [46,88,89]. These influences are particularly potent among adolescents and in households with a lower socioeconomic status, where access to healthy alternatives is limited and digital marketing strategies are more effective [45,89].

### 4.6. Adolescence: Consolidation and Vulnerability

Adolescence is a critical stage in the development of eating behaviors. Increased autonomy, peer influence and exposure to new social norms can shape eating preferences and practices [7,27,46]. During this period, the pursuit of novel experiences and sensitivity to reinforcement are linked to a greater preference for energy-dense foods and impulsive behaviors, particularly in environments where such foods are easily accessible [37,39].

However, this stage also offers opportunities for change. Interventions that promote food literacy, critical thinking toward marketing and participation in healthy school environments can support self-regulation and attenuate risky tendencies [27,74].

Integrating these findings into our narrative synthesis, we argue that early determinants of eating behaviors establish trajectories connecting biological and environmental influences, which are progressively consolidated from pregnancy to adolescence [23,49,87,90]. Conversely, continued exposure to environments that promote obesity, intensive digital marketing and food insecurity are associated with hedonistic patterns and poor self-regulation [84,91].

Figure 2 summarizes the evidence by developmental stage and level of determinant, mapping the developmental trajectory of these early-life influences. Rather than presenting isolated stages, this synthesis emphasizes a cumulative process where early biological and proximal factors build upon each other. This representation enables us to visualize the convergence of factors from an ecological and multiscale perspective, ultimately leading to the consolidated eating behaviors and dietary intake that characterize adolescence.

## 5. Mechanisms Linking Eating Behaviors and Diet Intake

Recent systematic reviews have revealed that specific eating behavior patterns are consistently associated with distinct dietary profiles in childhood and adolescence. Children and adolescents who exhibit food approach tendencies, such as higher food responsiveness or greater enjoyment of food, tend to consume more foods that are high in sugars and fats, but they also consume more fruit and vegetables. In contrast, those with more pronounced food avoidance traits, such as satiety responsiveness or food fussiness, generally exhibit lower overall food and energy intake, except for snacks, which are often consumed more frequently [28]. These findings emphasize the dual influence of approach and avoidance tendencies on eating habits during development.

### 5.1. Individual-Level Mechanisms

At an individual level, eating behaviors are the result of the combined influence of neurobiological, psychological and cognitive processes. It is not simply an automatic response to hunger. Rather, it emerges from the integration of homeostatic, hedonic and cognitive signals [32,39,40]. The homeostatic and hedonic systems are central to this regulation: the former is mediated by hypothalamic circuits and hormones such as leptin and ghrelin and seeks to maintain energy balance, while the latter is driven by dopamine and other reward neurotransmitters and promotes the pursuit of pleasure from highly palatable foods [35,92,93]. Together, these biological mechanisms may help explain the differential susceptibility to overeating observed during childhood and adolescence [10,49].

These biological mechanisms co-occur with temperamental and environmental factors in shaping appetite self-regulation [32,80]. Exposure to responsive parenting practices and structured environments is associated with responsiveness to internal hunger and satiety cues [17,48,94]. Conversely, coercive use of food as a reward or punishment, or early exposure to highly palatable foods, is associated with reduced sensitivity to these cues, fostering a greater hedonic response associated with impulsive eating patterns and reduced self-control [8,15,93,95]. These dispositions, shaped by early experiences, contribute to the formation of relatively stable trajectories that persist into adolescence [7].

### 5.2. Family-Level Mechanisms

At the family level, parenting practices and the emotional climate during mealtimes play a central role in shaping children’s responsiveness to internal hunger and satiety cues [26]. Children who are exposed to responsive parenting and structured routines tend to develop better self-regulation skills. In contrast, coercive feeding practices, such as using food as a reward or punishment, and early exposure to highly palatable foods, are associated with altered sensitivity to internal cues, potentially reinforcing hedonic responses and impulsive eating [49,69,96,97,98].

In households affected by parental stress, food insecurity or a lack of time, there is often a greater reliance on energy-dense, low-nutrient foods, resulting in a worsening of eating habits [44,45,99,100]. These family dynamics co-occur with individual characteristics to either strengthen or weaken appetite self-regulation.

### 5.3. Community and Socio-Cultural Mechanisms

At the community and socio-cultural levels, marketing influences can either reinforce or mitigate these mechanisms. The availability and marketing of highly palatable foods can shape hedonic circuits of pleasure and reward, thereby increasing exposure to stimuli that override physiological satiety signals [85,101]. This phenomenon is particularly pertinent during adolescence, when brain plasticity and the pursuit of social reinforcement amplify vulnerability to these stimuli [37,98].

Furthermore, the internalizing of social norms, parental modelling and emotional experiences during mealtimes shape emotional associations with food, which can perpetuate both protective and risky patterns [7,15].

Therefore, to understand the mechanisms linking eating behavior and diet intake, a biopsychosocial perspective is required, in which neurobiological, familial and structural factors act interdependently throughout development.

## 6. Synthesis of Evidence and Conceptual Framework

The scientific evidence gathered in this review reveals a consistent association between early-life determinants of eating behaviors and eating habits during childhood and adolescence. Longitudinal studies conducted in diverse settings suggest that appetitive traits shaped in early life are linked to subsequent dietary intake, and vice versa. Moreover, food environments, including conditions at home, school, and digital settings, are also associated with these longitudinal patterns. Although the directionality of these associations varies across study designs and contexts, the literature repeatedly highlights appetite self-regulation and responsive parenting practices as central processes underpinning healthier developmental trajectories [7,18,48,69].

As summarized above, there is consistent evidence that responsive feeding practices during early childhood and authoritative parenting styles are key determinants of appetite self-regulation and healthier dietary habits [42,43,95]. Supportive family dynamics, including shared meals and consistent modelling, further strengthen children’s ability to recognize internal hunger and satiety cues, thereby promoting lasting food preferences [7,44,102,103]. Results from the HELENA study confirm that regular family meals are associated with higher dietary quality and better psychosocial outcomes among European adolescents [104]. Together, these findings emphasize the pivotal role of early family environments in shaping eating behaviors throughout development.

Beyond the family context, available literature suggests the influence of structural determinants. School-based nutrition programs and community interventions that improve access to fruit, vegetables, and drinking water have consistently demonstrated a positive impact on eating habits [16,82,88]. However, environments dominated by highly palatable foods and pervasive digital marketing continue to drive impulsive and hedonic eating patterns, particularly during adolescence [39,89,101].

Nevertheless, important knowledge gaps and conflicting findings persist in the literature. There is substantial heterogeneity in the methodological approaches used across studies, which often leads to contradictory results regarding the magnitude of early-life-effects, such as breastfeeding or specific parenting styles. The most notable inconsistencies arise from two sources. First, eating behaviors measurements predominantly rely on parental self-reports, which sometimes contradict objective observational data. Second, a wide range of scales is used to quantify dietary intake and nutritional patterns. The scarcity of longitudinal research, especially in low-income settings, coupled with the limited cross-cultural validation of the instruments, restricts the generalizability of the findings and highlights the necessity for a more inclusive and equitable approach.

In summary, the strongest evidence is concentrated in three areas: responsive eating in early childhood, self-regulatory parenting practices, and the monitoring of school and digital food environments. Figure 2 provides a comprehensive synopsis of the interplay between these structural determinants, biological factors, and early family influences across the developmental trajectory.

This representation illustrates how these diverse layers, from innate predispositions to policy environments, converge over time. The pathways shown in Figure 2 reflect hypothesized associations derived from heterogeneous evidence and should be interpreted as a conceptual framework. They illustrate the cumulative process that leads to consolidated eating behaviors, habits, and dietary intake in adolescence, rather than as uniform causal effects.

## 7. Practical and Policy Implications

The synthesized evidence suggests that eating behavior trajectories are shaped from a very early stage, opening a potential window for preventive interventions. An important approach for promoting appetite self-regulation and addressing dysregulated eating behaviors appears to be the implementation of responsive feeding practices during breastfeeding and complementary feeding [23,42,43,70].

Within the family context, interventions that bolster parenting abilities, such as dietary education programs, teaching caregivers to recognize hunger and fullness food cues and to offer a variety of foods without coercion, have been linked to improvements in children’s eating habits [41,95]. Family meals free from digital distractions and characterized by positive parenting may also support the internalization of healthy habits [33,103].

At a structural level, schools and communities represent pivotal environments [105]. The implementation of nutritional standards in school cafeterias and measures to limit the sale of energy-dense foods are associated with reduced exposure to low-nutrient foods and the potential promotion of healthy habits during childhood [21,83,106]. Urban policies that facilitate access to fresh, healthy and affordable foods, such as community markets and school gardens, may also contribute to promoting dietary diversity and food equity [45,82,87].

At a broader policy level, several national strategies have been implemented with the aim of shaping children’s eating behaviors, not only by modifying the food environment but also by potentially reducing exposure to food cues that drive appetite and food choice [107,108]. The implementation of taxes on sugar-sweetened beverages has been linked to changes in the consumption patterns of children, including reduced intake of sugar-sweetened beverages and shifts in preferences towards lower-sugar alternatives [109,110,111,112,113]. This phenomenon might suggest a possible shift in the motivators behind beverage selection, indicating potential changes in the psychological underpinnings of choice [114].

Furthermore, the implementation of enhanced school meal standards in the United States and the United Kingdom has been followed by increased acceptance of fruits, vegetables, and whole grains among children, alongside reduced reliance on energy-dense snacks during school hours [105,115]. Other strategies, such as front-of-pack warning labels and restrictions on the marketing of unhealthy foods to children, particularly in Chile, have been associated with reduced exposure to persuasive food cues which may stimulate food responsiveness [116,117,118]. These measures have been associated with healthier purchasing patterns among families [107,117]. Collectively, these strategies suggest that modifying environmental cues could effectively promote healthier eating habits in childhood. Finally, future policy strategies could consider prioritizing the regulation of digital marketing targeting minors, particularly on social media platforms, where exposure to persuasive food advertising remains high [88,89].

## 8. Strengths and Limitations

This review provides a comprehensive biopsychosocial synthesis of eating behaviors and dietary intake from infancy to adolescence, addressing modern challenges like digital food environments. However, as an interpretivist narrative review, this work is inherently subjective. The literature selection was critical and iterative rather than exhaustive, allowing for a flexible interpretation that may involve selection bias [30].

Furthermore, the significant heterogeneity in study designs, ranging from parent-reports to experimental assessments of eating behavior, limits our ability to draw definitive causal inferences. Finally, while we emphasize the bidirectionality between appetitive traits and environmental factors, there is still a lack of longitudinal data regarding modern influences on appetite self-regulation, such as social media.

## 9. Conclusions

Eating behavior develops from the earliest stages of life through the combined influence of biological factors, family experiences and broader social contexts. Evidence synthesized in this review indicates that early-life influences are associated with patterns that consolidate during childhood and adolescence and may extend into adulthood, contributing to long-term dietary quality and individual differences in appetite self-regulation.

Rather than acting in isolation, our interpretive model emphasizes that responsive feeding practices, early exposure to healthy foods and structured family environments emerge as recurrent features of healthier developmental trajectories, while sustained exposure to obesogenic environments is consistently linked to less adaptive eating patterns and reduced sensitivity to internal satiety cues across developmental stages.

Overall, these findings suggest the need for a preventive, multisectoral approach that moves beyond individual-level interventions. Coordinated actions across the home, school, community and digital environments, embedded within broader policies addressing social inequalities, appear essential to support a healthier relationship with food and healthy development throughout life course.

## 10. Future Directions and Research Gaps

Despite the prioritization of longitudinal evidence in this review, it must be acknowledged that a significant portion of research on eating behaviors remains cross-sectional. This methodological landscape limits the ability to establish temporal or causal directions between early determinants and later diet quality, as most findings describe associations at a single point in time rather than developmental trajectories.

Future research is needed for greater standardization in the measurement of appetitive traits and parenting food practices. The variety of instruments used and the reliance on self-reporting can introduce bias and make it difficult to compare results from different studies. Incorporating validated observational measures and mixed-methods approaches would help to capture the complexity of eating behavior more accurately.

Future research should move beyond descriptive associations to better characterize the mechanisms linking early determinants, eating behaviors, and dietary intake across the life course. This will require a stronger emphasis on intervention studies and quasi-experimental designs capable of informing causal pathways, particularly in relation to parenting practices and early food environments.

In parallel, advancing the biological understanding of appetite regulation will require integrating multi-omics approaches beyond genomics alone. While most existing evidence relies on genetic association studies, future work incorporating transcriptomics, epigenomics, metabolomics, proteomics, lipidomics, and the gut microbiome may help elucidate biological pathways underlying eating behaviors, identify intermediate biomarkers linking environmental exposures to appetite regulation, characterize dynamic biological processes across sensitive developmental windows, and inform the timing, tissue specificity, and potential reversibility of these mechanisms across the life course.

Methodologically, future studies should also address confounding and potential reverse causation by adopting using repeated-measures designs, baseline adjustment for appetite traits, cross-lagged and sibling-comparison approaches, and the use of natural experiments where feasible. Together, these advances would strengthen causal inference and support the development of more targeted and developmentally informed prevention strategies.

Finally, dynamic systems models and equity-focused, intergenerational interventions represent a promising way to translate evidence into transformative public policies. Integrating biological, behavioral, and structural dimensions will enable healthier, more accessible, and more sustainable food environments to be created for children and adolescents.

## Data Availability

No new data were created or analyzed in this study. Data sharing is not applicable to this article.

## Abbreviations

The following abbreviations are used in this manuscript:

BMI	Body Mass Index
*FTO*	Fat mass and obesity-associated gene
*MC4R*	Melanocortin-4 receptor
SES	Socioeconomic status
SSB	Sugar-sweetened beverages
TEDS	Twins Early Development Study
